# Angiotensin‐Converting Enzyme 2 (SARS‐CoV‐2 receptor) expression in human skeletal muscle

**DOI:** 10.1111/sms.14061

**Published:** 2021-10-04

**Authors:** Mario Perez‐Valera, Miriam Martinez‐Canton, Angel Gallego‐Selles, Victor Galván‐Alvarez, Miriam Gelabert‐Rebato, David Morales‐Alamo, Alfredo Santana, Saul Martin‐Rodriguez, Jesus Gustavo Ponce‐Gonzalez, Steen Larsen, Jose Losa‐Reyna, Ismael Perez‐Suarez, Cecilia Dorado, David Curtelin, Juan Jose Gonzalez‐Henriquez, Robert Boushel, Jostein Hallen, Pedro de Pablos Velasco, Jorge Freixinet‐Gilart, Hans‐Christer Holmberg, Jorn W. Helge, Marcos Martin‐Rincon, Jose A.L. Calbet

**Affiliations:** ^1^ Department of Physical Education University of Las Palmas de Gran Canaria Las Palmas de Gran Canaria Spain; ^2^ Research Institute of Biomedical and Health Sciences (IUIBS) University of Las Palmas de Gran Canaria Las Palmas de Gran Canaria Spain; ^3^ Complejo Hospitalario Universitario Insular‐Materno Infantil de Las Palmas de Gran Canaria Clinical Genetics Unit Las Palmas de Gran Canaria Spain; ^4^ Center of Healthy Ageing Department of Biomedical Sciences University of Copenhagen Copenhagen Denmark; ^5^ Clinical Research Centre Medical University of Bialystok Bialystok Poland; ^6^ Department of Mathematics University of Las Palmas de Gran Canaria Las Palmas de Gran Canaria Spain; ^7^ School of Kinesiology Faculty of Education The University of British Columbia Vancouver British Columbia Canada; ^8^ Department of Physical Performance The Norwegian School of Sport Sciences Oslo Norway; ^9^ Department of Endocrinology and Nutrition Hospital Universitario de Gran Canaria Doctor Negrín Las Palmas de Gran Canaria Spain; ^10^ Department of Thoracic Surgery Hospital Universitario de Gran Canaria Dr. Negrín Las Palmas de Gran Canaria Spain; ^11^ Department of Physiology and Pharmacology Biomedicum C5 Karolinska Institute Stockholm Sweden; ^12^ Department of Health Sciences Luleå University of Technology Luleå Sweden

**Keywords:** COVID‐19, ACE2, obesity, cardiorespiratory fitness, exercise, VO2max, sex differences, biopsies

## Abstract

The study aimed to determine the levels of skeletal muscle angiotensin‐converting enzyme 2 (ACE2, the SARS‐CoV‐2 receptor) protein expression in men and women and assess whether ACE2 expression in skeletal muscle is associated with cardiorespiratory fitness and adiposity. The level of ACE2 in *vastus lateralis* muscle biopsies collected in previous studies from 170 men (age: 19–65 years, weight: 56–137 kg, BMI: 23–44) and 69 women (age: 18–55 years, weight: 41–126 kg, BMI: 22–39) was analyzed in duplicate by western blot. VO_2_max was determined by ergospirometry and body composition by DXA. ACE2 protein expression was 1.8‐fold higher in women than men (*p* = 0.001, *n* = 239). This sex difference disappeared after accounting for the percentage of body fat (fat %), VO_2_max per kg of legs lean mass (VO_2_max‐LLM) and age (*p* = 0.47). Multiple regression analysis showed that the fat % (β = 0.47) is the main predictor of the variability in ACE2 protein expression in skeletal muscle, explaining 5.2% of the variance. VO_2_max‐LLM had also predictive value (β = 0.09). There was a significant fat % by VO_2_max‐LLM interaction, such that for subjects with low fat %, VO_2_max‐LLM was positively associated with ACE2 expression while as fat % increased the slope of the positive association between VO_2_max‐LLM and ACE2 was reduced. In conclusion, women express higher amounts of ACE2 in their skeletal muscles than men. This sexual dimorphism is mainly explained by sex differences in fat % and cardiorespiratory fitness. The percentage of body fat is the main predictor of the variability in ACE2 protein expression in human skeletal muscle.

## 1 INTRODUCTION

Like previous coronaviruses, severe acute respiratory syndrome coronavirus 2 (SARS‐CoV‐2) penetrates human cells by binding to the angiotensin‐converting enzyme 2 (ACE2).^1^, ^2^ A high level of ACE2 protein expression may facilitate cell infection and vice versa,^1^ and tissue differences in ACE2 expression could explain part of the pathophysiology of the disease.^3^, ^4^ ACE2 mRNA overexpression has been reported in patients with comorbidities (hypertension, diabetes, obesity, ischemic cardiomyopathy, among others) who are at higher risk of a more severe COVID‐19.^5^, ^6^ Male sex, ageing, and low cardiorespiratory fitness and physical inactivity have also been associated with increased severity and mortality from COVID‐19.^7^, ^8^, ^9^, ^10^, ^11^, ^12^ However, it remains unknown whether sex, age, cardiorespiratory fitness, and body composition are associated with different levels of ACE2 protein expression in humans.

ACE2 mRNA is ubiquitously expressed in human tissues and especially abundant in the lungs and testis, and a little less abundant in the kidney, heart, digestive tract and skeletal muscle.^13^, ^14^, ^15^, ^16^ ACE2 is necessary to counteract the canonical renin‐angiotensin system (RAS).^17^ A misbalance between angiotensin‐converting enzyme and ACE2 facilitates myocardial inflammation and fibrosis,^18^ and in mice, skeletal muscle ACE2 deficiency has been related to muscle weakness.^19^ However, it remains unknown whether ACE2 expression in skeletal muscle is associated with exercise capacity in humans. Besides, even though SARS‐CoV‐2 targets skeletal muscle,^20^ and myalgia is a common symptom of the disease,^8^ there is almost no information regarding the ACE2 protein expression levels in human skeletal muscle.^21^, ^22^ Sex hormones may influence several components of the RAS and related serum peptidases,^23^, ^24^ implying that menstrual status should also be considered.

Therefore, the aims of this study were: (1) to determine the levels of ACE2 protein expression in human skeletal muscle; (2) to ascertain whether the levels of ACE2 protein expression show sexual dimorphism in humans; and (3) to assess whether ACE2 protein expression in human skeletal muscle is associated with cardiorespiratory fitness and adiposity.

We hypothesized that ACE2 protein expression in skeletal muscle would be inversely associated with cardiorespiratory fitness, adiposity and age, and that men would present greater levels of ACE2 protein expression than premenopausal women.

## 2 METHODS

### 2.1 Study design and participants

This is a cross‐sectional study using muscle biopsies obtained from 170 men and 69 women who participated in previous studies,^25^, ^26^, ^27^, ^28^, ^29^, ^30^, ^31^ including some of the participants in a European research project^32^ named METAPREDICT, and some subjects involved in ongoing research projects.^33^, ^34^ All subjects were non‐smokers, 105 of them being healthy university students with different physical activity levels and the other 134 were mainly sedentary with overweight or obesity. In the group with overweight or obesity, 27 subjects had hypertension (i.e., systolic blood pressure >130 or diastolic >80 mmHg; 4 of them treated with diuretics, 3 with inhibitors/blockers of the RAS, and 1 with a calcium antagonist), two subjects were on statins, and one had type 2 diabetes (treated with diet and exercise). In addition, all women were premenopausal since this was an inclusion criterion, and three of them were taking oral contraceptives. All participants volunteered to participate in the corresponding studies and signed written informed consent after receiving complete information regarding the aims of the studies and potential side effects of the procedures. All experiments were performed per the Declaration of Helsinki after ethical approval.

### 2.2 Main procedures

All volunteers were requested to avoid strenuous physical activity for 48 h and to refrain from caffeinated and alcoholic beverages for 24 h preceding preceding all tests and the muscle biopsies. Before the start of the experiments, subjects were familiarized with the exercise tests. After that, their anthropometric characteristics were registered, and their body composition determined by dual‐energy X‐ray absorptiometry (Lunar iDXA, General Electric, WI, USA), as previously reported.^35^ This was followed by an incremental cycle ergometer exercise test (Lode Corival/Excalibur Sport, Groningen, The Netherlands) until exhaustion to determine their maximal oxygen consumption (VO_2_max) and maximal power output (Wmax). The incremental exercise test was adapted to the volunteers´ characteristics with load increments eliciting exhaustion in no less than 6 min and no more than 20 min.^36^ Oxygen uptake (VO_2_) during all exercise tests was measured by open‐circuit indirect calorimetry with metabolic carts (Vyntus, Jaeger‐CareFusion, Hoechberg, Germany; Vmax N29, Sensormedics, Yorba Linda, CA, USA; COSMED, Rome, Italy; and Jaeger Oxycon Pro, Viasys Healthcare, Hoechberg, Germany) operated in breath‐by‐breath mode. The gas analyzers were calibrated immediately before each test according to the instructions of the manufacturers. Respiratory variables were analyzed breath‐by‐breath and averaged every 20 s during the incremental exercise tests.^37^ The highest 20‐s averaged VO_2_ was taken as the VO_2_max and expressed per kg of leg lean mass (VO_2_max‐LLM). VO_2_max and DXA data were not obtained in 24 of the 170 males included in the study. A complete set of data was obtained for the rest of the variables assessed.

### 2.3 Muscle biopsies

The biopsies were performed following a 10–12 h overnight fast. Biopsies were taken from the middle portion of the m. *vastus lateralis* using Bergstrom's technique with suction. After disinfection of the skin, 1–2 ml local anesthetic (Lidocaine 2% without epinephrine) was injected into the skin and subcutaneous tissue, taking care not to inject below the superficial fascia. Ten minutes later, a 6–7 mm incision was made, and the biopsy needle inserted 2 cm into the muscle belly. The muscle sample (~100 mg) was dissected free of debris and fat tissue and immediately frozen in liquid nitrogen at −80°C until analyzed.

### 2.4 Protein extraction and western blotting

Whole skeletal muscle lysates were prepared as previously reported,^26^ and protein concentration quantified using the bicinchoninic acid assay^33^ to ensure equal sample concentration. In brief, ~10 mg of muscle was ground by stainless steel balls during one minute in a Mikro‐Dismembrator S (Sartorius, Goettingen, Germany) and immediately homogenized in urea lysis buffer (6 M urea, 1% SDS) with 50X Complete protease and 10× PhosSTOP phosphatase inhibitors (Roche, Mannheim, Germany). Subsequently, the lysate was centrifuged for 12 min at 25 200 g at 16°C. The resulting supernatant containing the protein fraction was diluted with electrophoresis loading buffer (160 mM Tris‐HCl, pH 6.8, 5.9% SDS, 25.5% glycerol, 15% β‐mercaptoethanol‐ bromophenol blue). An equal amount of total protein from each sample (10 μg) was loaded onto the gels following antibody linearity optimization tests. All samples were run in duplicate and averaged. In all gels, four lanes were loaded with the same internal control (non‐interventional human muscle) to allow for normalization to the mean value of the control sample to compensate for variability between gels. Then, gels were electrophoresed with SDS‐PAGE using the system of Laemli^38^ and proteins were transferred onto the polyvinylidene fluoride (PVDF) membranes for protein blotting (Bio‐Rad Laboratories, Hercules, CA, USA). Membranes were blocked for one hour in 5% non‐fat dry milk powder (blotting‐grade blocker, Bio‐Rad Laboratories) in Tris‐buffered saline containing 0.1% Tween 20 (TBS‐T) (Blotto blocking buffer) and incubated overnight at 4°C with the primary antibody (catalogue no. ab108252, Abcam, Cambridge, UK), diluted 1:2000 in Blotto blocking buffer. This was followed by washes and 1‐hour incubation at room temperature with the secondary antibody (HRP‐conjugated goat anti‐rabbit IgG, catalogue no. 111‐035‐144, Jackson ImmunoResearch Laboratories Inc., West Grove, PA, USA), diluted 1:5000 in Blotto blocking buffer, and subsequent chemiluminescent visualization with Clarity™ Western ECL Substrate (Bio‐Rad Laboratories) using the ChemiDoc™ Touch Imaging System (Bio‐Rad Laboratories). Densitometry band quantification was performed with the Image Lab^©^ software 5.2.1 (Bio‐Rad Laboratories). In order to verify equal loading and transfer efficiency, membranes were stained with Reactive Brown 10 (Sigma‐Aldrich).

### 2.5 Statistical analysis

The Gaussian distribution of variables was determined using the Shapiro–Wilk test, and statistical tests in non‐normally distributed variables were run with logarithmically transformed data. Men and women general characteristics were compared using an unpaired t‐test. Sex differences in ACE2 protein expression levels were assessed with ANCOVA, using body fat percentage (fat %), VO_2_max‐LLM and age as covariates. The contribution of fat %, VO_2_max‐LLM and age to ACE2 protein expression were further tested using multiple linear regression analysis with fat % × VO_2_max‐LLM interaction. Since a significant fat % × VO_2_max‐LLM interaction was observed, the Johnson‐Neyman procedure was applied to identify the point where the relationship between the independent and the outcome variable transitioned from being statistically significant to nonsignificant or vice versa. Values are reported as the mean ± standard deviation (SD). Statistical significance was set at *p* < 0.05. All statistical analyses were performed using IBM SPSS Statistics version 26 for Mac (IBM, NY, US) and R version 4.0.5 (R Foundation for Statistical Computing, Vienna, Austria).

## 3 RESULTS

### 3.1 Study population

Two hundred and thirty‐nine participants from 18 to 65 years old were analyzed in this study. The descriptive characteristics of the study population are reported in Table 1. Women had a higher percentage of body fat, and lower VO_2_max than men, even when expressed per kg of legs’ lean mass. A negative association was observed between VO_2_max expressed per kg of body weight and ACE2 protein expression in the whole population (*r* = −0.25, *p *< 0.001, N = 215).

**TABLE 1:** Baseline characteristics of the study population

	Men (*n* = 170)			Women (*n* = 69)			*p*
	Mean ± SD	Range		Mean ± SD	Range		
Age (years)	29.1 ± 9.7	18.6	65.2	33.0 ± 10.5	18.2	54.9	0.005
Weight (kg)	87.1 ± 17.0	55.9	136.9	81.5 ± 17.3	41.3	126.1	0.012
Height (cm)	177.8 ± 7.2	161.0	198.3	163.6 ± 6.4	150.0	180.0	<0.001
BMI (kg.m^−2^)	25.5 ± 9.6	22.7	43.6	30.3 ± 5.7	22.1	38.7	<0.001
Body fat (%)^a^	27.1 ± 9.8	7.7	45.6	42.5 ± 8.1	21.5	54.6	<0.001
Total lean mass (kg)	60.4 ± 6.7	45.6	79.4	43.6 ± 6.0	28.8	58.2	<0.001
VO_2_max (ml.min^−1^)^a^	3453 ± 554	2019	4834	2150 ± 403	1219	3462	<0.001
VO_2_max (ml.kg^−1^.min^−1^)^a^	40.4 ± 10.5	20.6	61.9	27.5 ± 7.5	12.6	47.6	<0.001
VO_2_max (ml.kg LM^−1^.min^−1^)^a^	57.4 ± 8.6	38.0	75.7	49.6 ± 8.3	28.8	68.9	<0.001
VO_2_max (ml.kg LLM^−1^.min^−1^)^a^	162.6 ± 30.9	106.3	253.2	137.9 ± 24.1	82.8	201.4	<0.001

Note

Data presented as mean ± standard deviation. *p*‐values presented correspond to comparisons between men and women.

Abbreviations: LM, Lean mass; LLM, Legs’ lean mass; SD, standard deviation.

^a^ Body composition and VO_2_max was not determined in 24 men, and therefore N = 146 for these variables in men.

### 3.2 ACE2 expression: sex differences

**FIGURE 1:**
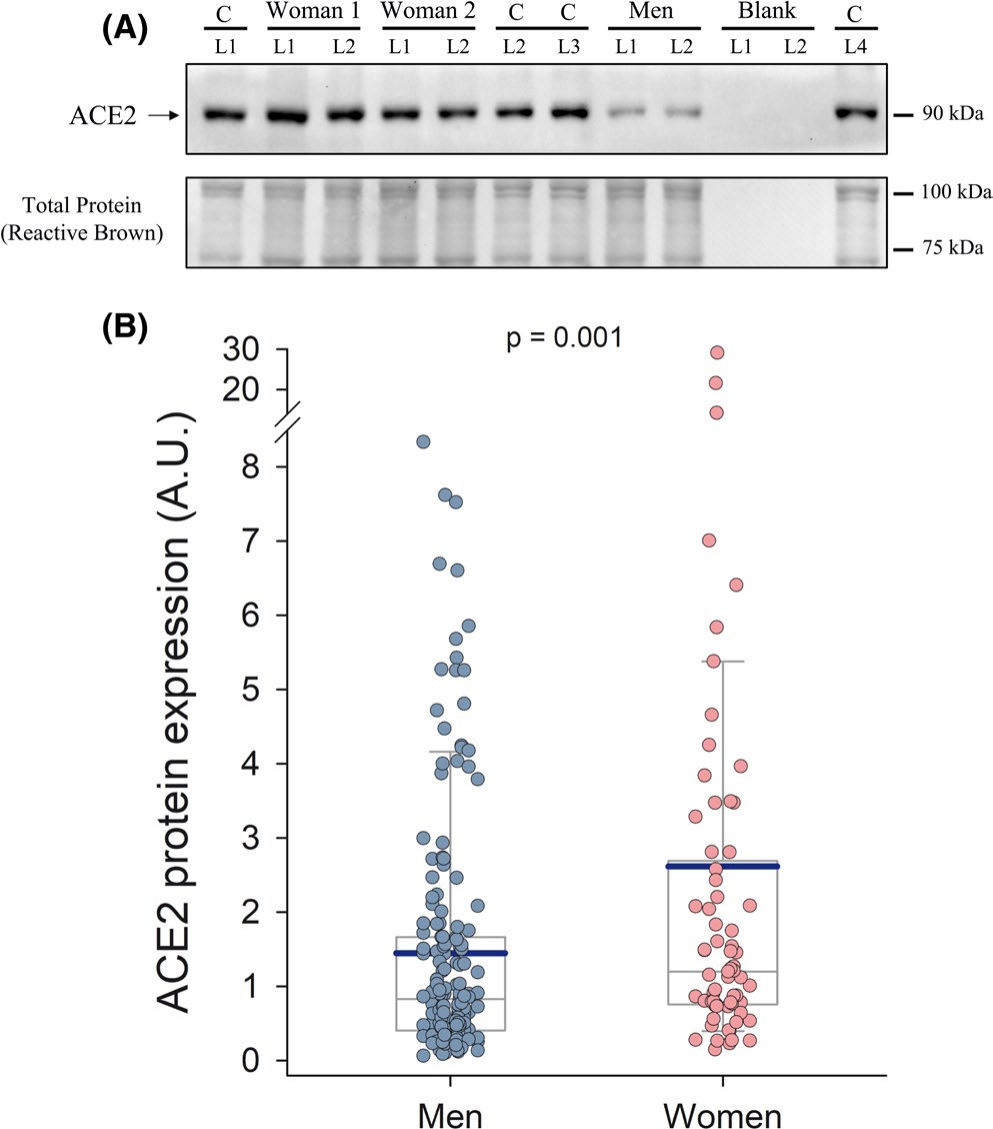
Skeletal muscle ACE2 protein expression levels in men and women covering a wide range of age, fitness, and body composition: (A) Representative immunoblot images and total amount of protein loaded (Reactive Brown Staining) from two women and one man participating in the study. All experimental samples were run in duplicate, while a control human sample (non‐experimental) was included onto each gel to allow normalization and loading control. C, control non‐experimental sample; L, gel lane for a particular sample or blank. Estimated molecular weights are indicated by arrows on the side of the blot. (B) Box and whisker plots of ACE2 skeletal muscle protein expression in all men (*n* = 170) and women (*n* = 69) included in the study (arbitrary units, A.U.). The extremes of the whiskers represent the limits of the 5th and 95th percentiles, respectively; the thin and thick horizontal lines inside the boxes correspond to the mean and median values, respectively; and the lower and upper limits of the box delimit the 1st and 3rd quartiles, respectively. The statistical analysis was performed with logarithmically transformed data. *p*‐value = 0.001 for the difference between men and women (unpaired two‐tailed *t*‐test) with all data included in the analysis and *p* = 0.007 after removing the two women with higher ACE2 values

**FIGURE 2:**
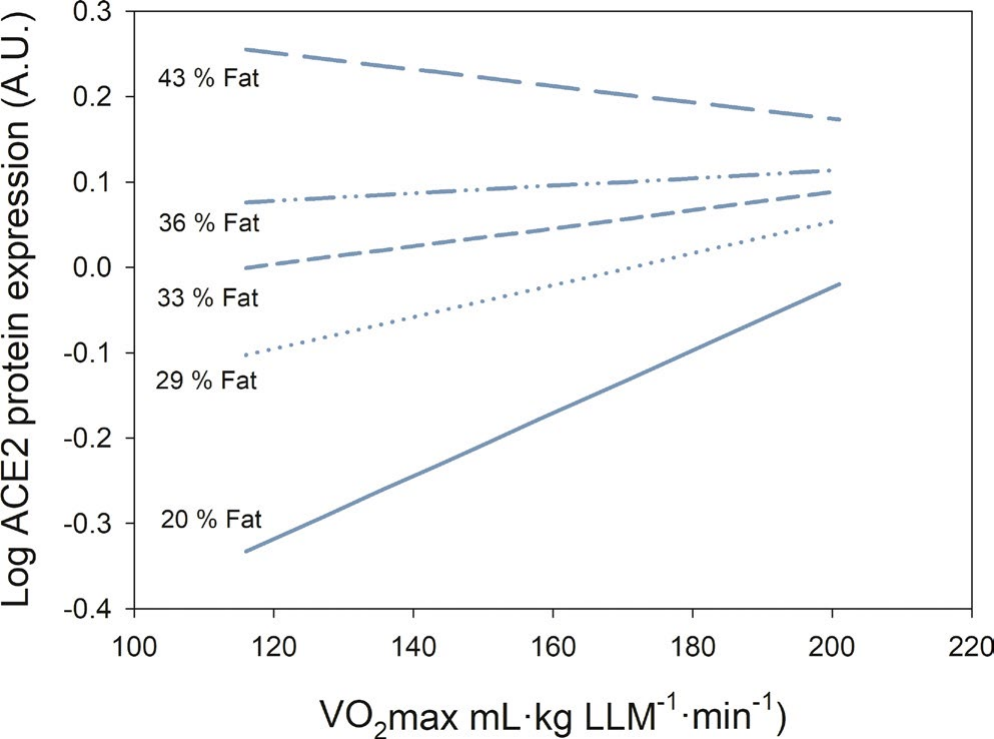
Skeletal muscle ACE2 protein expression and cardiorespiratory fitness. Each straight line represents the linear relationship between the logarithm of the level of ACE2 expression in the human *vastus lateralis* skeletal muscle and maximal oxygen uptake (VO_2_max, expressed as mL of O_2_ per kg of lean mass of the lower extremities (LLM)), for the percentages of body fat indicated. N = 215 (146 men and 69 women). This graph was created using the regression model reported in Table 2

**FIGURE 3:**
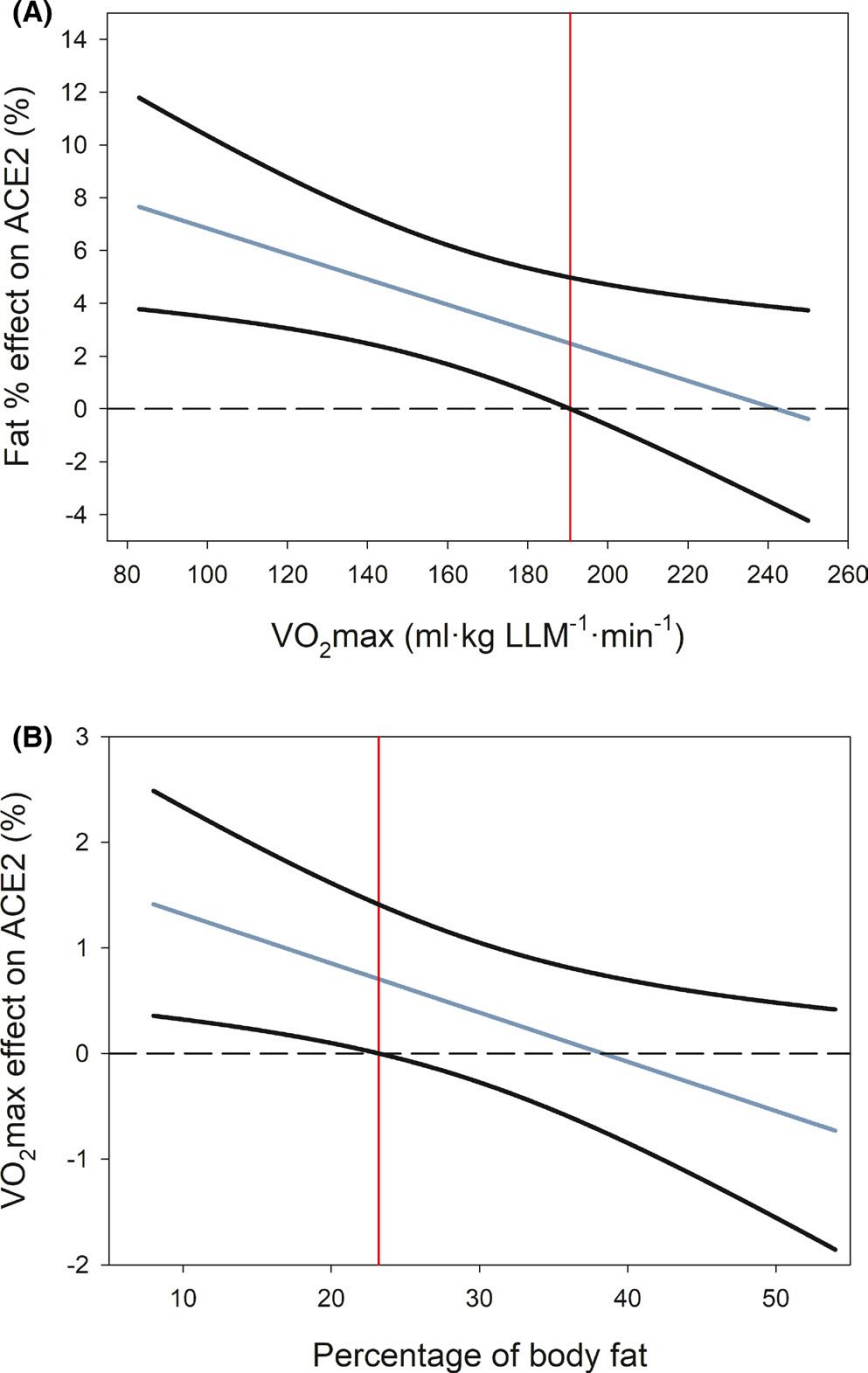
Johnson‐Neyman analysis depicting the moderator effects of the percentage of body fat and cardiorespiratory fitness on ACE2 protein expression in human skeletal muscle. (A) Moderator effect of VO_2_max on the linear relationship between the percentage of body fat (fat %) and ACE2 protein expression. The black lines depict the 95% confidence interval, and the red line indicates the value of VO_2_max (190.6 ml·kg LLM^−1^·min^−1^) above which the moderator effect is not statistically significant. Below this value, for a given level of VO_2_max, ACE2 expression increases in the percentage indicated in the “Y” axis per each unit of increase of fat %. (B) Moderator effect of fat % on the linear relationship between VO_2_max and ACE2 protein expression. The black lines depict 95% confidence interval, and the red line indicates the value of fat % (23.2%), above which the moderator effect is not statistically significant. Below this value, for a given level of fat %, ACE2 expression increases in the percentage indicated in the “Y” axis per each unit of increase in VO_2_max

ACE2 protein expression was 1.8‐fold higher in women than men (*p* = 0.001, N = 239) (Figure 1). However, this sex difference disappeared after accounting for fat % (*p* = 0.72), VO_2_max‐LLM (*p* = 0.15), fat % and VO_2_max‐LLM (*p* = 0.57), or fat %, VO_2_max‐LLM and age (*p* = 0.47) as covariates. ACE2 protein expression was positively associated with fat % in both sexes (*r* = 0.18 and 0.37, *p* = 0.03 and =0.002, in men and women, respectively). In the whole group of subjects, multiple regression analysis showed that the fat % (β = 0.47) is, out of the 4 predictors studied (age, sex, adiposity, VO_2_max), the main predictor of the variability in ACE2 protein expression in skeletal muscle (Table 2), explaining 5.2% of the variance. VO_2_max‐LLM also had predictive value (β = 0.09); however, the predictive value of fat % was 5.3‐fold higher than that of VO_2_max‐LLM. There was a significant fat % by VO_2_max‐LLM interaction, explaining 2.5% of the variance, such that for subjects with low fat %, VO_2_max‐LLM was positively associated with ACE2 expression while as fat % increased, the association between VO_2_max and ACE2 disappears (Figures 2 and 3). The Johnson‐Neyman analysis indicated that VO_2_max‐LLM moderates the association between fat % and ACE2 expression. For VO_2_max‐LLM values below 190.6 ml.kg LLM^−1^.min^−1^, the positive influence of fat % on ACE2 expression was steeper the lower the VO_2_max‐LLM (Figure 3A). Likewise, for fat % below 23.2, ACE2 expression increased with VO_2_max‐LLM more markedly the lower the fat % (Figure 3B).

**TABLE 2:** Factors predicting ACE2 protein expression in human skeletal muscle

Predictor	Estimate	SE	T	*p*	Standardized Estimate (β)	*R* ^2^
Intercept^a^	−1.65187	0.53853	−3.067	0.002		
Sex	−0.0965	0.08283	−1.165	0.245	−0.218	0.022
Fat %	0.04898	0.01444	3.393	<0.001	0.473	0.075
VO_2_ max (ml.kg LLM^−1^)	0.00772	0.00287	2.692	0.008	0.089	0.082
Age (years)	−0.00292	0.00331	−0.883	0.378	−0.068	0.084
Fat % × VO_2_max (ml.kg LLM^−1^)	−0.000202	0.000084	−2.399	0.017	−0.166	0.109

Note

LLM: leg lean mass; ACE2 was logarithmically transformed; N = 215, due to lack of measurements of VO_2_max and LLM in 24 men; Sex: Men = 1, Women = 2.

^a^ Represents reference level (Men = 1).

## 4 DISCUSSION

In the present investigation, the ACE2 protein expression levels in skeletal muscle have been determined for the first time in a large sample of men and women, covering a wide range of adiposity, cardiorespiratory fitness, and age. We have shown that premenopausal women express larger amounts of ACE2 protein than men of similar age. This sex dimorphism is fully accounted for by differences in adiposity and cardiorespiratory fitness between men and women, with fat % playing a predominant role. An interaction was observed between fat % and VO_2_max, such that in subjects with low fat %, there is a positive linear association between ACE2 and VO_2_max. However, as fat % increases, the positive slope of the relationship between ACE2 and VO_2_max is attenuated to reach values close to zero in subjects with high levels of adiposity, who present the larger amount of ACE2. The higher ACE2 expression in individuals with higher adiposity may be necessary to counteract the canonical renin‐angiotensin system (RAS),^39^ whose activity is augmented with obesity and ageing.^40^, ^41^, ^42^, ^43^

### 4.1 Women have greater levels of ACE2 than men

ACE2 reduces the levels of angiotensin II by converting its precursor (Ang I) into Ang 1–9 and by transforming Ang II into Ang 1–7. Both peptides Ang 1–7 and 1–9 have vasodilatory, anti‐inflammatory, antioxidant, antifibrotic, and antithrombotic effects in tissues and contribute to reducing blood pressure.^17^, ^39^, ^44^, ^45^ Thus, ACE2 has a protective function and could be one of the factors contributing to the lower prevalence of cardiovascular disease in premenopausal women.^46^ Although a higher expression of ACE2 may raise the risk of SARS‐CoV‐2 infection, epidemiological data indicate that the risk of infection seems similar in both sexes.^47^ The latter contrasts with the higher severity and mortality of COVID‐19 in men than women,^11^, ^47^, ^48^ attributed to sex differences in the innate antiviral defence.^11^, ^48^ A higher expression of ACE2 in skeletal muscle could predispose women to myalgias and fatigue, which are more prevalent in women than men.^49^, ^50^

The present findings contrast with the lack of sex differences in serum ACE2 activity in humans aged 41–70 years old.^24^ Therefore, the sexual dimorphism here reported may vary depending on the menstrual status and sex hormone concentrations in blood, as observed for other components of the RAS.^23^, ^24^

### 4.2 ACE2 expression is positively associated with the percentage of body fat

In our study population, the percentage of body fat is the strongest predictor of ACE2 protein expression in skeletal muscle in both sexes. COVID‐19 disease severity and mortality increase with BMI in both sexes.^51^, ^52^ Although the underlying mechanisms for this association remain unknown, it has been suggested that the low‐grade inflammation and metabolic derangements of obesity may facilitate COVID‐19 infection and severity.^53^

The fact that obesity is associated with increased expression of ACE2 could facilitate cellular invasion in patients with obesity.^1^ Moreover, since docking of SARS‐CoV‐2 to ACE2 leads to internalization and degradation of ACE2, this could cause a misbalance between ACE2/RAS facilitating vasoconstriction, inflammation, fibrosis, and thrombosis. Likely, patients with overweight or obesity need to express higher amounts of ACE2 in their tissues to counteract their overactive RAS, and therefore, being more sensitive to the harmful effects of a potential reduction of tissular ACE2 levels after SARS‐CoV‐2 infection.

### 4.3 ACE2 expression is positively associated with VO_2_max‐LLM in lean subjects

This study has shown that subjects with lower cardiorespiratory fitness, expressed as VO_2_max per kg of body mass, present higher levels of ACE2 in the skeletal muscle. However, this association is mediated by the inverse relationship that exists between VO_2_max per kg of body mass and fat %. Interestingly, our regression model indicates that ACE2 protein expression is positively associated with VO_2_max‐LLM in lean but not in obese subjects, showing the predominant effect of adiposity in obese subjects (Figure 2). It is essential to highlight that in the present investigation, VO_2_max has been normalized to lean mass of the lower extremities, i.e., our measurements of VO_2_max are independent of adiposity. This implies that the reported lower severity of COVID‐19 in patients with higher cardiorespiratory fitness^10^ is likely mediated by the inverse relationship between VO_2_max and adiposity, since the higher the body fat percentage, the lower the VO_2_max per kg of body weight.

Since ACE2 could contribute to exercise hyperemia by reducing the levels of angiotensin II, from a mechanistic perspective, the observed positive relationship between VO_2_max‐LLM and ACE2 seems reasonable since VO_2_max is mainly limited by O_2_ delivery during whole‐body exercise.^54^ Thus, regular exercise, which is usually associated with lower levels of adiposity, could upregulate ACE2 in skeletal muscle and thereby enhance exercise hyperemia. Although the reason why ACE2 expression in skeletal muscle is increased in obesity is currently unknown, we presume that this is a counterregulatory response to the overactivation of classical RAS in obesity. To test this hypothesis, ACE2 expression in skeletal muscle of patients with obesity should be determined before and after pharmacological inhibition of RAS.^55^

### 4.4 Strengths and limitations

The main strengths of this study are the large number of subjects included with skeletal muscle biopsies and the assessment of VO_2_max and body composition with state‐of‐art‐techniques, including men and women with large differences in age and fitness levels. Another strength is that all muscle expression measurements were performed in duplicate to reduce variability. The main limitation of this study relates to its cross‐sectional design.

In summary, this study shows that premenopausal women express a higher amount of ACE2 in their skeletal muscles. This sexual dimorphism is mainly explained by sex differences in the percentage of body fat. The present investigation reveals that skeletal muscle ACE2 protein expression is positively associated with the levels of adiposity in humans. Further research is needed to verify whether the observation made in skeletal muscle also extends to other tissues and how exercise influences ACE2 protein expression levels in skeletal muscle and other tissues.

### 4.5 Perspectives

The impact that regular exercise may have on ACE2 expression in skeletal muscle must be studied with specific exercise training programs. Since ACE2 expression has tissue specificity, the observed female predominance of ACE2 expression in human skeletal muscle should be confirmed in other tissues, including the airways.^56^, ^57^ Future studies should determine the physiological implications of the variability observed in skeletal muscle ACE2 expression, including the assessment of the different components of the ACE2/Ang 1‐7/Mas signaling pathway and the associated vasodilatory peptides.

## CONFLICT OF INTEREST

The authors declare that they have no conflict of interest.

## AUTHOR CONTRIBUTIONS

The contributions of the authors are as follows: MPV, MMC, MMR, and JALC contributed to conception and design of the study; all co‐authors contributed to collection, analysis, and interpretation of data; MPV, MMR, and JALC drafted the manuscript; all co‐authors critically evaluated and contributed to the manuscript. All authors have approved the final version of the manuscript.

## Data Availability

Deidentified participant data are available from the senior author (ORCID: 0000‐0002‐9215‐6234) on reasonable request for research purposes.
